# Pathophysiology and Diagnosis of Pulmonary Hypertension Due to Left Heart Disease

**DOI:** 10.3389/fmed.2018.00174

**Published:** 2018-06-06

**Authors:** Athanasios Charalampopoulos, Robert Lewis, Peter Hickey, Charlotte Durrington, Charlie Elliot, Robin Condliffe, Ian Sabroe, David G. Kiely

**Affiliations:** Sheffield Pulmonary Vascular Disease Unit, Royal Hallamshire Hospital, Sheffield Teaching Hospitals NHS Foundation Trust, Sheffield, United Kingdom

**Keywords:** pulmonary hypertension, left heart disease, heart failure with preserved ejection fraction, left ventricular diastolic dysfunction, right heart catheterization

## Abstract

Pulmonary hypertension due to left heart disease (PH-LHD) is the most common type of pulmonary hypertension, although an accurate prevalence is challenging. PH-LHD includes PH due to systolic or diastolic left ventricular dysfunction, mitral or aortic valve disease and congenital left heart disease. In recent years a new and distinct phenotype of “combined post-capillary and pre-capillary PH,” based on diastolic pulmonary gradient and pulmonary vascular resistance, has been recognized. The roles of right ventricular dysfunction and pulmonary vascular compliance in PH-LHD have also been elucidated recently and they appear to have significant clinical implications. Echocardiography continues to play a seminal role in diagnosis of PH-LHD and heart failure with preserved LV ejection fraction, as it can identify valve disease and help to distinguish PH-LHD from pre-capillary PH. Right, and occasionally left heart catheterization, remains the gold-standard for diagnosis and phenotyping of PH-LHD, although Cardiac Magnetic Resonance Imaging is emerging as a useful alternative tool in non-invasive diagnostic and prognostic assessment of PH-LHD. In this review, the latest evidence for more recent advances will be discussed, including the role of fluid challenge and exercise during cardiac catheterization to unravel occult post-capillary and the role of vasoreactivity testing. The use of many or all of these diagnostic techniques will undoubtedly provide key information about sub-groups of patients with PH-LHD that might benefit from medical therapy previously considered to be only suitable for pulmonary arterial hypertension.

## Introduction

Pulmonary hypertension due to left heart disease (PH-LHD) is the most common type of pulmonary hypertension (PH). The prevalence of PH in patients with heart failure varies significantly with diagnostic criteria from 25 to 83% (1–4). PH-LHD is defined by post-capillary hemodynamics at right heart catheterization (RHC); that is a mean pulmonary arterial pressure ≥25 mmHg and a mean pulmonary arterial wedge pressure (PAWP) > 15 mmHg. PAWP is a surrogate marker of left atrial pressure (LAP). An elevated LAP causing PH can occur in systolic and/or diastolic left ventricular (LV) dysfunction and in left-sided valvular disease. In the most recent clinical classification of PH (5) two additional etiologies of PH-LHD have been recognized: PH due to congenital or acquired left ventricular outflow tract obstruction and pulmonary vein stenosis. In the same guidelines, a new PH phenotype of “combined pre-capillary and post-capillary PH” (Cpc-PH) has been introduced on the grounds of a diastolic pressure gradient (DPG)-the difference between diastolic pulmonary arterial pressure and mean PAWP-equal or higher than 7 mmHg (Table 1). This new term came to replace the older “PH out of proportion to LHD.” Although, pathophysiology of Cpc-PH is not entirely clear, a chronic elevation of LAP due to longstanding LHD is believed to cause a profound pulmonary artery remodeling and pulmonary vascular resistance (PVR) rise, which is not usually found in isolated post-capillary PH. Patients with Cpc-PH seem to be in the middle of a spectrum of which pre-capillary PH and isolated post-capillary PH are the two extremes, regarding their clinical and echocardiographic characteristics (6).The prevalence of Cpc-PH amongst patients with systolic and diastolic heart failure is believed to be within 12 and 14% (7). An adequate understanding of pathophysiology along with an accurate diagnosis and differentiation of PH-LHD from pre-capillary PH, such as pulmonary arterial hypertension (PAH) and chronic thromboembolic PH (CTEPH), are of paramount importance to select the appropriate treatment for the patient.

**Table 1:** Definitions.

Pre-capillary PH	Mean PAP ≥ 25 mmHg, mean PAWP ≤ 15 mmHg
Isolated post-capillary PH	Mean PAP ≥ 25 mmHg, mean PAWP >15 mmHg, DPG < 7 mmHg and/or PVR ≤ 3 Wood units
Combined pre-capillary and post-capillary PH	Mean PAP ≥ 25 mmHg, mean PAWP >15 mmHg, DPG ≥ 7 mmHg and/or PVR > 3 Wood units

*PAP, pulmonary artery pressure; PAWP, pulmonary artery wedge pressure; DPG, diastolic pressure gradient; PVR, pulmonary vascular resistance. Modified from 2015 ESC/ERS Pulmonary Hypertension Guidelines (5)*.

## Pathophysiology

**Figure 1:**
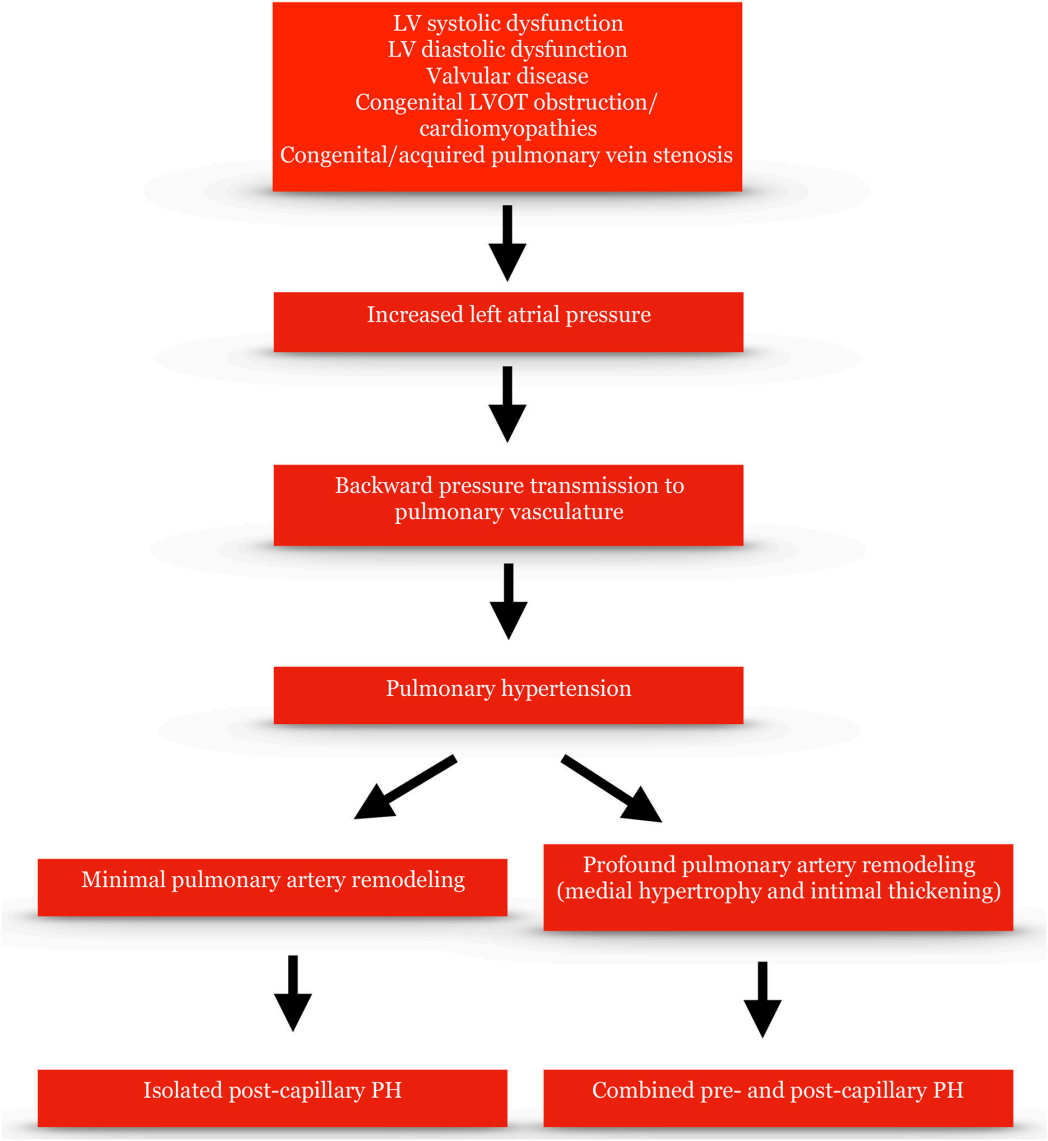
Pathophysiological mechanisms of pulmonary hypertension due to left heart disease.

The pathophysiological hallmark of PH-LHD is elevated LAP. LV systolic and diastolic dysfunction as well as aortic and/or mitral valve stenosis and/or regurgitation can raise left ventricular filling pressure and subsequently LAP over a period of time. LAP can then be transmitted backwards via pulmonary veins to the pulmonary vasculature leading to pulmonary arterial intimal thickening and medial hypertrophy and PH (Figure 1). Compliance in the pulmonary vasculature, unlike in the systemic circulation, is more evenly distributed across the pulmonary bed and the distal vessels are responsible for most of it (8). Hence, compliance is mostly determined by PVR. The relationship between PVR and compliance is an inverse hyperbolic one. “Passive” left-sided elevated pressures shift the hyperbole leftwards leading to an additional decline of compliance for a given PVR and thus enhanced pulmonary wave reflections which return during ventricular systole and increase the systolic (but not the diastolic) pulmonary artery pressure (9). In a recent study, pulmonary arterial compliance (or capacitance) defined as the ratio of stroke volume to pulmonary pulse pressure was the best predictor of mortality in PH-LHD associated with heart failure with preserved left ventricular ejection fraction (HFpEF) (10).

### The right ventricle (RV)

The first compensatory mechanism of the RV to the elevated pulmonary pressure is hypertrophy. Thus, the RV can adapt with a 4- to 5-fold increase in myocardial contractility, a process which is described pathophysiologically as right ventricular-pulmonary artery (RV-PA) “coupling.” In the next progressive stage the RV starts dilating, wall stress according to LaPlace's law increases, imbalance between oxygen demand and supply and RV ischemia occurs, contractility declines, the RV fails to maintain cardiac output and inevitably “uncoupling” and decompensation begins. The development of tricuspid (TR) and pulmonary regurgitation due to annular dilatation leads to RV volume overloading and decreases stroke volume even further. RV-PA coupling has been estimated by echocardiography as the ratio of tricuspid annular plane systolic excursion (TAPSE) to pulmonary artery systolic pressure (PASP) and a cut-off limit <0.36 has been shown to have prognostic value (11). Coupling has also been estimated by a hybrid method combining RHC and cardiac magnetic resonance (CMR) as the ratio of pulmonary artery effective elastance, which is a measure of arterial load, to the maximum end-systolic RV elastance, which is an index of contractility (12).

Up until recently the impact of PH-LHD on RV function had been underestimated. In recent years, however, its significance and prognostic value has been highlighted. RV systolic dysfunction assessed by echocardiography has been found in at least 1/5 of patients with HFpEF and potentially up to 30–50% (13). In addition, a decrease of TAPSE by 5 mm was associated with a 26% mortality rise (13), while in another study a decrease of RV fractional area change by 7% with a 2.2-fold increase of all-cause mortality after adjustment for pulmonary pressures in patients with HFpEF (14).

### Ventricular interdependence and comorbidities

Other parameters such as ventricular interdependence may contribute to decreased cardiac output and heart failure. As RV pressure increases and interventricular dyssynchrony is established, the interventricular septum bows toward the LV moving “paradoxically” and leading to a decrease of LV diastolic filling and a further drop of stroke volume. In addition, atrial fibrillation, coronary artery disease via right coronary or circumflex artery stenosis leading to reduced RV oxygen supply, chronic obstructive pulmonary disease, obstructive sleep apnea, diabetes, and obesity, all comorbidities frequently seen in patients with LHD may increase pulmonary pressures and further compromise RV function.

### The role of DPG

DPG has been considered to be a more accurate indicator of pulmonary vascular remodeling in LHD than transpulmonary gradient (the difference between mean pulmonary artery pressure and mean PAWP [TPG]), as it is theoretically not influenced by the variability of cardiac output or the impact of LAP on pulmonary vascular compliance. In a retrospective large study in patients with PH-LHD an elevated DPG ≥ 7 mmHg was correlated with worse median survival compared with a DPG < 7 mmHg and a high TPG > 12 mmHg (15). In the same study, in a small number of patients high DPG was also correlated with greater pathological pulmonary vascular remodeling compared to patients with high TPG but normal DPG. Two further studies, retrospective as well, in patients who had undergone orthotopic heart transplantation and in patients with unexplained cardiomyopathy did not confirm the prognostic value of DPG (16, 17). In a more recent study the risk of death in Cpc-PH and isolated post-capillary PH after adjustment of relevant covariates was similar, however the researchers identified 75 common exonic single-nucleotide polymorphisms between Cpc-PH and pre-capillary PH in pathways pertinent with cell structure, extracellular matrix and immune function (6).

## Diagnosis

### Risk factors, clinical symptoms, and signs

Features of metabolic syndrome such as obesity, systemic hypertension, diabetes and dyslipidemia, are more prevalent in patients with PH-LHD than PAH. Researchers have shown that two or more of these features were present in 94.1% of PH-LHD patients vs. only 34.3% of PAH (18). The findings were confirmed in another study, which showed that older age, the presence of systemic hypertension and coronary artery disease are more frequent in PH-LHD and can best differentiate from PAH along with other echocardiographic and hemodynamic parameters (19). In the same study there was a similarly high female predominance in the PH-LHD and PAH groups but more pronounced than in the HFpEF without PH group. In addition to these risk factors, the prevalence of permanent atrial fibrillation in PH-LHD seems to be significantly higher than in patients with PAH and risk factors for LHD (20).

The symptoms and clinical signs of patients with PH-LHD are these of heart failure. Exertional dyspnea, orthopnea, and/or paroxysmal nocturnal dyspnea, anginal chest pain typically on exertion, palpitations, dry cough, pre-syncope and/or syncope, hepatomegaly, ascites, elevated jugular venous pressure, and peripheral edema are the most common ones. Right ventricular heave, a loud pulmonary component of the second heart sound and a pansystolic murmur of tricuspid regurgitation may also be present. In a retrospective study which compared patients with PAH with or without cardiovascular comorbidities to PH-LHD, patients with PH-LHD were more likely to have peripheral edema, whilst there was a trend toward a worse functional class after adjustment for age but no difference in 6-min walking test distance (20). Patients with PH-LHD usually have normal resting oxygen saturations with a possible drop on exercise, unless if they also have respiratory comorbidity or a right-to-left shunt.

### Chest X-ray, ECG, and pulmonary function test

The chest X-ray may show signs of cardiomegaly and enlarged pulmonary artery branches. Pleural effusions may be seen more frequently in PH-LHD than PAH, although they are not uncommon in severe PAH as well (21).

Left QRS axis deviation, atrial fibrillation and signs of left ventricular hypertrophy are more likely on ECG in PH-LHD than in pre-capillary PH, whereas right axis, sinus rhythm, R wave dominant in lead V1, ST-T segment depression, and T-wave inversion in the right precordial and inferior leads are more frequently seen in pre-capillary PH. Right bundle branch block does not appear to differentiate the two conditions (20, 22). These ECG findings have been recently incorporated in predictive score models to discriminate non-invasively between PH-LHD and PAH (23, 24).

It is well-known that pulmonary alveolar diffusion is impaired in patients with congestive heart failure. Gas transfer factor for carbon monoxide (TLco) is reduced in this population compared to controls (25). In addition, TLco and end-tidal CO_2_ have been found to be lower in PAH patients compared to PH-LHD (26, 27). In the ASPIRE registry, mean TLco in the PAH group was 55 vs. 62% predicted in the PH-LHD one (26).

### Natriuretic peptides

Brain natriuretic peptide (BNP) and its N-terminal fragment NT-proBNP can both be elevated in heart failure with reduced LV ejection fraction and HFpEF. They can be secreted from the cardiomyocytes of both ventricles due to stretching from volume overload. The upper limit of normal for BNP is 35 pg/mL and for NT-proBNP 125 pg/mL (28). Natriuretic peptides may be elevated in elderly people, atrial fibrillation and renal impairment without heart failure. They can also be disproportionally low in obese patients. In addition, they cannot differentiate between pre-capillary PH and PH-LHD as they may be raised in both conditions. Their role in patients' follow-up, assessment of response to treatment and detection of progression of the underlying disease as well as their prognostic role can be very important.

### Echocardiography

Echocardiography plays a seminal role in the detection of signs of PH-LHD. It can diagnose LV systolic and diastolic dysfunction, aortic and mitral valve disease, LV outflow tract obstruction, restrictive and hypertrophic cardiomyopathy and constrictive pericarditis. In all these conditions, LV diastolic dysfunction usually occurs and may lead to PH-LHD. The estimation of PASP, which raises the suspicion of PH when elevated, derives from applying continuous Doppler and the modified Bernoulli equation: *4 x peak TR velocity (TRV)*^2^ + *estimated right atrial pressure*. Right atrial pressure can be estimated from the size and collapsibility during respiration/sniff of the inferior vena cava on the subcostal view (29). According to the current ESC/ERS guidelines a threshold of peak TRV higher than 2.8 m/s is considered suspicious of PH with or without the presence of other echocardiographic signs of PH and risk factors for PH (5). However, peak TRV is angle and flow-dependent and it can frequently be under- or overestimated (30). Peak TRV should be measured from several different acoustic windows and views, while in atrial fibrillation an average of measurements from the view with the highest velocities should be taken and only a well-defined, spade-shaped, dense spectral profile should be measured. In cases with no or trivial TR when TRV cannot accurately be estimated, mean pulmonary artery pressure can be calculated instead from the peak velocity of a pulmonary regurgitant jet at the beginning of diastole or from the acceleration time (time from onset to peak) of the forward flow at the right ventricular outflow tract (29).

Diagnosing LV diastolic dysfunction with echocardiography sometimes can be challenging. Four parameters have been recently included in an algorithm to determine diastolic dysfunction: an average (of septal and lateral) E/e' > 14, a septal e' < 7 cm/s or lateral <10 cm/s, a TRV > 2.8 m/s and a left atrial volume index >34 ml/m^2^. If <50% of these parameters are present, diastolic function is normal, if >50%, there is diastolic dysfunction and when exactly 50%, diastolic function is indeterminate (31). Other echocardiographic signs such as mitral “L” velocity >20 cm/s (ongoing LV filling in mid-diastole due to markedly delayed LV relaxation), mitral inflow E/A wave velocity ratio and E wave deceleration time, as well as a higher diastolic wave velocity than the systolic one in the pulmonary vein Pulsed Doppler profile may also be useful (31). Unlike PH-LHD, significant LV diastolic dysfunction in PAH is very unlikely. In a small study, 88% of PAH patients demonstrated grade I (mild) diastolic dysfunction and 10% normal diastolic function (32).

Left atrial dilatation is considered to be one of the most useful indicators of LHD. However, in the presence of atrial fibrillation may not always represent elevated LAP. In atrial fibrillation, an average E/e' ≥11 instead of 14 should be used as a marker of diastolic dysfunction (31). Researchers in the past have described conditions such as severe mitral regurgitation with a dilated left atrium but normal LAP. Left atrial compliance seems to play a pivotal role in these cases (33). The absence of mid-systolic “notching” in the right ventricular outflow tract Pulsed Doppler envelope has also been shown to be highly predictive of a PVR < 3 Wood units and a PAWP >15 mmHg (34). On the other hand, a PASP > 70 mmHg is more likely to represent PAH than PH-LHD (35). The severity of TR cannot differentiate between PH-LHD and PAH. In a prospective cohort of patients with PAH and PH-LHD, 45% had mild, 34% moderate, and 21% severe TR. The mechanisms causing TR were annular dilatation, RV remodeling-tethering and inadequate tricuspid valve leaflet area (36). Finally, a RV end-diastolic diameter to LV end-diastolic diameter ratio (measured in the 4-chamber apical view at the tips of the atrioventricular valves) < 1 and the absence of pericardial effusion seem to be more likely in PH-LHD than PAH (20). Table 2 summarizes the distinct echocardiographic characteristics in pre-capillary PH and PH-LHD. An echocardiographic score based on LA dimension, mid-systolic notching and E/e' to differentiate between PH-LHD and PAH has been proposed (37). More recently another echocardiographic score, which seems to be useful in discriminating between isolated and Cpc-PH as well, has been published (38).

**Table 2:** Echocardiographic features likely to be present in pre- and post-capillary pulmonary hypertension.

**Pre-capillary PH**	**PH-LHD**
Normal sized or small LV cavity	Normal sized or dilated LV cavity
No LV hypertrophy	LV hypertrophy
Preserved LVEF	Variable LVEF
Normal sized or small left atrium	Dilated left atrium
Grade I LV diastolic dysfunction or normal LV diastolic function	≥ Grade II LV diastolic dysfunction
Presence of mid-systolic notching	Absence of mid-systolic notching
RV/LV ratio > 1	RV/LV ratio < 1
PASP > 70 mmHg	Typically PASP < 70 mmHg
Pericardial effusion	No pericardial effusion
No mitral and/or aortic valve disease	Mitral and/or aortic valve disease

*PH-LHD, pulmonary hypertension due to left heart disease; LV, left ventricle; LVEF, left ventricular ejection fraction; RV/LV ratio, right ventricular to left ventricular end-diastolic diameter ratio; PASP, pulmonary artery systolic pressure. Modified from Roberts JD and Forfia PR. Pulm Circ. 2011;1:160-181*.

### CMR

CMR may play an important role in making diagnosis and identifying prognosis of PH in general and PH-LHD in particular. Especially in cases where visualization or accuracy of measurements is limited, CMR should be strongly considered. CMR remains the gold standard imaging modality for RV assessment (39). Beyond RV size and function, CMR can quantify the curvature of interventricular septum and more recently pulmonary arterial stiffness, which may detect early stages of pulmonary vascular remodeling (40, 41). In the ASPIRE registry, patients with PH-LHD had lower RV mass, a better-preserved cardiac function and less gadolinium hinge-point enhancement compared to pre-capillary PH (42). In addition, CMR is an excellent tool for myocardial tissue characterization and with the use of late gadolinium enhancement it can diagnose infiltrative cardiomyopathies e.g., amyloidosis, hypertrophic cardiomyopathy, as well as systolic and diastolic LV dysfunction. CMR can also play a significant role in diagnosis of congenital heart diseases, which may be related to both PAH and PH-LHD.

### Predictive non-invasive score models

Over the last few years, score models to assess the pre-RHC probability of PH-LHD have been created aspiring to avoid potentially unnecessary catheterizations. Bonderman et al. proposed a model based on RV strain on ECG and BNP (23). Jacobs et al. suggested a risk model including history of LHD, the sum of S deflection in V1 and R deflection in V6 on the ECG, left atrial dilatation and left valve disease worse than mild on echocardiogram (24). Finally, Richter et al. suggested a prediction score for elevated PAWP based on age >68 years, BMI > 30 kg/m^2^, absence of RV enlargement and presence of left atrial dilatation on echocardiography (43).

### RHC-fluid challenge-exercise-vasoreactivity testing

The gold standard method for the diagnosis of PH-LHD remains RHC. All pulmonary pressures should be measured at end-expiration with the patient breathing spontaneously. The mean PAWP has been better correlated with mean LAP than the LV end-diastolic pressure (LVEDP) in patients with LV disease, except for very high PAWP over 25 mmHg (44, 45). However, a LVEDP > 15 mmHg on left heart catheterization can be used to define PH-LHD, when an accurate PAWP is not feasible (e.g., in chronic thromboembolic disease). An accurate PAWP may be challenging to obtain. A waveform consistent with atrial waveform, a PAWP ≤ diastolic pulmonary arterial pressure, blood easily aspirated and highly oxygenated in the wedge position and the placement of the tip of the catheter in West zone 3 (at the lung base, where pulmonary artery and venous pressure exceeds pulmonary alveolar pressure, which represents lung areas with the greatest blood flow rates), below left atrial level, can eliminate technical difficulties. Mitral regurgitation may cause large “v” waves that can be mistakenly interpreted as an elevated PAWP. This can be accounted for by reading the PAWP at the time of the “a” wave. Patients with PH-LHD tend to have a higher mean right atrial pressure, cardiac output and right ventricular end-diastolic pressure, as well as lower PVR compared with PAH (19, 20).

Patients with clinical and echocardiographic features suggestive of PH-LHD may sometimes have pre-capillary PH, especially if they have been previously heavily diuresed. Fluid challenge, which is the rapid intravenous administration of a high volume of normal saline, is believed to be helpful to unpick cases of occult PH-LHD with resting pre-capillary hemodynamics and unmask LV diastolic dysfunction. The current ESC/ERS PH guidelines do not recommend fluid loading at RHC due to lack of standardization as well as the theoretical risk of a rapid significant PAWP elevation to result in pulmonary edema. Three studies with fluid challenge in PH patients have been conducted since the publication of these guidelines though. In the first one, the infusion of 500 mL of saline within 5–10 min led to the reclassification of 22.2% of patients as occult PH-LHD (defined as a mean PAWP > 15 mmHg), while in another small study in patients with systemic sclerosis a similar percentage of patients with pre-capillary baseline hemodynamics were found to have a LVEDP > 15 mmHg post-infusion of 500 mL within 5–10 min (46, 47). In the most recent one, rapid infusion of saline at a rate of 7 mL/kg reclassified 8% of patients with no PH and 6% of patients with pre-capillary PH at baseline. A cut-off limit of PAWP ≥ 18 mmHg was used in the latter study (48). In all three studies, fluid loading was safe. Of note, even in healthy subjects a rapid fluid infusion will lead to a PAWP > 15 mmHg in a percentage >60%, however with a larger than 500 mL volume of saline (49). Likewise, exercise during RHC may unmask diastolic dysfunction in patients with suspected HFpEF and a baseline PAWP < 15 mmHg. Exercise can be performed on a supine or upright bike (the latter would be feasible when the RHC is done from the neck or the arm) or with leg lifting. In normal subjects and athletes, an increase of PAWP up to 25 mmHg in a broadly linear fashion with cardiac output has been shown (50). However, significant increases of PAWP at a cardiac output <10 L/min is uncommon in normal individuals. Exercise during RHC seems to be particularly helpful in obese patients with a borderline, between 12 and 15 mmHg, PAWP (51).

According to the ESC/ERS guidelines vasoreactivity testing is only recommended in patients with suspected idiopathic, familial, or drug-induced PAH (5). In the context of PH-LHD vasodilator challenge can be used in the evaluation of patients for listing for cardiac transplantation, especially those with a TPG > 12–15 mmHg and/or PVR > 3 Wood units. The preferable vasodilator agent is inhaled nitric oxide. An increase of PAWP post-nitric oxide may be seen but pulmonary edema is very rare.

### Treatment

PAH targeted therapies act on three distinct pathophysiological pathways. The first one is the nitric oxide-soluble guanylate cyclase (sGC)-cGMP pathway which includes drugs like phosphodiesterase type 5 inhibitors (Sildenafil, Tadalafil) and stimulators of cGC like Riociguat. The second one is the endothelin-1 pathway with drugs like endothelin receptor antagonists (Bosentan, Ambrisentan, and Macitentan) and the third is the prostacyclin pathway with prostacyclin analogs (Epoprostenol, Treprostinil, Iloprost) and an oral selective IP prostacyclin-receptor agonist (Selexipag). These drugs have proven to be efficacious in PAH and CTEPH patients in several randomized, controlled, double-blind trials. On the other hand, trials of the same drugs in patients with PH-LHD have not shown any benefit and some of them have also been related with increased mortality (52–54). In the recent SIOVAC trial (55), where Sildenafil was tested vs. placebo in patients with post-capillary PH and history of correction of valvular heart disease, median PVR in the Sildenafil group was 3.4 Wood units, TPG 16 mmHg, but DPG only 2 mmHg. Of note, the study did not meet its primary end-point. Further trials need to be conducted in patients with Cpc-PH hemodynamics to answer the question about a possible efficacy of pulmonary vasodilator drugs in this specific PH phenotype.

## Conclusions

Up to now, no pulmonary vasodilator treatment has proven to be efficacious in PH-LHD. Some of the PAH drugs have even been detrimental when given in patients with LHD. Hence, it is apparent that the accurate identification of the major driver of PH in each patient is essential. In clinical practice the diagnosis and phenotyping of PH cannot be based on a single test, even if that test is the RHC, but rather on a combination of clinical data, imaging and hemodynamics. A better understanding of pathophysiology and further clinical trials are required to clarify whether Cpc-PH is a distinct clinical entity or part of a spectrum of PH phenotypes and identify potential treatments for the future.

## Author contributions

All authors listed have made a substantial, direct and intellectual contribution to the work, and approved it for publication.

### Conflict of interest statement

The authors declare that the research was conducted in the absence of any commercial or financial relationships that could be construed as a potential conflict of interest.

## References

[1] Lam CS Roger VL Borlaug BA Enders FT Redfield MM. Pulmonary hypertension in heart failure with preserved ejection fraction: a community-based study. J Am Coll Cardiol. (2009) 53:1119–26. 10.1016/j.jacc.2008.11.051 19324256 PMC2736110

[2] Leung CC Moondra V Catherwood E Andrus BW. Prevalence and risk factors of pulmonary hypertension in patients with elevated pulmonary venous pressure and preserved ejection fraction. Am J Cardiol. (2010) 106:284–6. 10.1016/j.amjcard.2010.02.039 20599017

[3] Damy T Goode KM Kallvikbacka-Bennett A Lewinter C Hobkirk J Nikitin NP. Determinants and prognostic value of pulmonary arterial hypertension in patients with chronic heart failure. Eur Heart J. (2010) 31:2280–90. 10.1093/eurheartj/ehq245 20693169

[4] Bursi F McNallan SM Redfield MM Nkomo VT Lam CS Weston SA. Pulmonary pressures and death in heart failure: a community study. J Am Coll Cardiol. (2012) 59:222–31. 10.1016/j.jacc.2011.06.076 22240126 PMC3258551

[5] Galiè N Humbert M Vachiery J-L Gibbs S Lang I Torbicki A. 2015 ESC/ERS Guidelines for the diagnosis and treatment of pulmonary hypertension: The Joint Task Force for the Diagnosis and Treatment of Pulmonary Hypertension of the European Society of Cardiology (ESC) and the European Respiratory Society (ERS): Endorsed by: Association for European Paediatric and Congenital Cardiology (AEPC), International Society for Heart and Lung Transplantation (ISHLT). Eur Heart J. (2016) 37:67–119. 10.1093/eurheartj/ehv317 26320113

[6] Assad TR Hemnes AR Larkin EK Glazer AM Xu M Wells QS Clinical and biological insights into combined post- and pre-capillary pulmonary hypertension. J Am Coll Cardiol. (2016) 68:2525–36. 10.1016/j.jacc.2016.09.942 27931609 PMC5157701

[7] Gerges M Gerges C Pistritto AM Lang MB Trip P Jakowitsch J. Pulmonary hypertension in heart failure: epidemiology, right ventricular function, and survival. Am J Respir Crit Care Med. (2015) 192:1234–46. 10.1164/rccm.201503-0529OC 26181215

[8] Saouti N Westerhof N Helderman F Marcus JT Stergiopulos N Westerhof BE. RC time constant of single lung equals that of both lungs together: a study in chronic thromboembolic pulmonary hypertension. Am J Physiol Heart Circ Physiol. (2009) 297:H2154–60. 10.1152/ajpheart.00694.2009 19801491

[9] Tedford RJ Hassoun PM Mathai SC Girgis RE Russell SD Thiemann DR. Pulmonary capillary wedge pressure augments right ventricular pulsatile loading. Circulation (2012) 125:289–97. 10.1161/CIRCULATIONAHA.111.051540 22131357 PMC3264431

[10] Al-Naamani N Preston IR Paulus JK Hill NS Roberts KE. Pulmonary arterial capacitance is an important predictor of mortality in heart failure with a preserved ejection fraction. JACC Heart Fail (2015) 3:467–74. 10.1016/j.jchf.2015.01.013 26046840 PMC4536851

[11] Guazzi M Bandera F Pelissero G Castelvecchio S Menicanti L Ghio S. Tricuspid annular plane systolic excursion and pulmonary arterial systolic pressure relationship in heart failure: an index of right ventricular contractile function and prognosis. Am J Physiol Heart Circ Physiol. (2013) 305:H1373–81. 10.1152/ajpheart.00157.2013 23997100

[12] Sanz J García-Alvarez A Fernández-Friera L Nair A Mirelis JG Sawit ST. Right ventriculo-arterial coupling in pulmonary hypertension: a magnetic resonance study. Heart (2012) 98:238–43. 10.1136/heartjnl-2011-300462 21917658

[13] Gorter TM Hoendermis ES van Veldhuisen DJ Voors AA Lam CS Geelhoed B. Right ventricular dysfunction in heart failure with preserved ejection fraction: a systematic review and meta-analysis. Eur J Heart Fail (2016) 18:1472–87. 10.1002/ejhf.630 27650220

[14] Melenovsky V Hwang SJ Lin G Redfield MM Borlaug BA. Right heart dysfunction in heart failure with preserved ejection fraction. Eur Heart J. (2014) 35:3452–62. 10.1093/eurheartj/ehu193 24875795 PMC4425842

[15] Gerges C Gerges M Lang MB Zhang Y Jakowitsch J Probst P. Diastolic pulmonary vascular pressure gradient: a predictor of prognosis in “out-of-proportion” pulmonary hypertension. Chest (2013) 1433:758–66. 10.1378/chest.12-1653 23580984

[16] Tampakakis E Leary PJ Selby VN De Marco T Cappola TP Felker GM. The diastolic pulmonary gradient does not predict survival in patients with pulmonary hypertension due to left heart disease. JACC Heart Fail (2015) 3:9–16. 10.1016/j.jchf.2014.07.010 25453535 PMC4289416

[17] Tedford RJ Beaty CA Mathai SC Kolb TM Damico R Hassoun PM. Prognostic value of the pre-transplant diastolic pulmonary artery pressure-to-pulmonary capillary wedge pressure gradient in cardiac transplant recipients with pulmonary hypertension. J Heart Lung Transplant (2014) 33:289–197. 10.1016/j.healun.2013.11.008 24462554 PMC3955214

[18] Robbins IM Newman JH Johnson RF Hemnes AR Fremont RD Piana RN. Association of the metabolic syndrome with pulmonary venous hypertension. Chest (2009) 136:31–36. 10.1378/chest.08-2008 19188551 PMC2716716

[19] Thenappan T Shah SJ Gomberg-Maitland M Collander B Vallakati A Shroff P. Clinical characteristics of pulmonary hypertension in patients with heart failure and preserved ejection fraction. Circ Heart Fail (2011) 4:257–65. 10.1161/CIRCHEARTFAILURE.110.958801 21411741

[20] Charalampopoulos A Howard LS Tzoulaki I Gin-Sing W Grapsa J Wilkins MR. Response to pulmonary arterial hypertension drug therapies in patients with pulmonary arterial hypertension and cardiovascular risk factors. Pulm Circ. (2014) 4:669–78. 10.1086/678512 25610602 PMC4278626

[21] Milne EN. Forgotten gold in diagnosing pulmonary hypertension: the plain chest radiograph. Radiographics (2012) 32:1085–7. 10.1148/rg.324125021 22786995

[22] McMurray JJ Carson PE Komajda M McKelvie R Zile MR Ptaszynska A. Heart failure with preserved ejection fraction: clinical characteristics of 4133 patients enrolled in the I-PRESERVE trial. Eur J Heart Fail. (2008) 10:149–56. 10.1016/j.ejheart.2007.12.010 18279770

[23] Bonderman D Wexberg P Martischnig AM Heinzl H Lang MB Sadushi R. A noninvasive algorithm to exclude pre-capillary pulmonary hypertension. Eur Respir J. (2011) 37:1096–103. 10.1183/09031936.00089610 20693249

[24] Jacobs W Konings TC Heymans MW Boonstra A Bogaard HJ van Rossum AC. Noninvasive identification of left-sided heart failure in a population suspected of pulmonary arterial hypertension. Eur Respir J. (2015) 46:422–30. 10.1183/09031936.00202814 25837029

[25] Smith AA Cowburn PJ Parker ME Denvir M Puri S Patel KR. Impaired pulmonary diffusion during exercise in patients with chronic heart failure. Circulation (1999) 100:1406–10. 10500041 10.1161/01.cir.100.13.1406

[26] Hurdman J Condliffe R Elliot CA Davies C Hill C Wild JM. ASPIRE registry: assessing the Spectrum of Pulmonary hypertension Identified at a referral centre. Eur Respir J. (2012) 39:945–55. 10.1183/09031936.00078411 21885399

[27] Hemnes AR Pugh ME Newman AL Robbins IM Tolle J Austin ED. End tidal CO_2_ tension: pulmonary arterial hypertension vs pulmonary venous hypertension and response to treatment. Chest (2011) 140:1267–73. 10.1378/chest.11-0155 21622547 PMC4694178

[28] Ponikowski P Voors AA Anker SD Bueno H Cleland JGF Coates AJS. 2016 ESC Guidelines for the diagnosis and treatment of acute and chronic heart failure. Eur Heart J. (2016) 37:2129–200. 10.1093/eurheartj/ehw128 27206819

[29] Rudski LG Wyman WL Afilalo J Hua L Handschumacher MD Chandrasekaran K. Guidelines for the echocardiographic assessment of the right heart in adults: a report from the american society of echocardiography. endorsed by the european association of echocardiography, a registered branch of the European Society of Cardiology, and the Canadian Society of Echocardiography. J Am Soc Echocardiogr. (2010) 23:685–713. 10.1016/j.echo.2010.05.010 20620859

[30] Fisher MR Forfia PR Chamera E Housten-Harris T Champion HC Girgis RE. Accuracy of Doppler echocardiography in the hemodynamic assessment of pulmonary hypertension. Am J Respir Crit Care Med. (2009) 179:615–21. 10.1164/rccm.200811-1691OC 19164700 PMC2720125

[31] Nagueh SF Smiseth OA Appleton CP Byrd BF III Dokainish H Edvardsen T. Recommendations for the evaluation of left ventricular diastolic function by echocardiography: an update from the American Society of Echocardiography and the European Association of Cardiovascular Imaging. Eur Heart J Cardiovasc Imaging (2016) 17:1321–60. 10.1016/j.echo.2016.01.011 27422899

[32] Tonelli AR Plana JC Heresi GA Dweik RA. Prevalence and prognostic value of left ventricular diastolic dysfunction in idiopathic and heritable pulmonary arterial hypertension. Chest (2012) 141:1457–65. 10.1378/chest.11-1903 22207680 PMC3367485

[33] Braunwald E Awe W. The syndrome of severe mitral regurgitation with normal left atrial pressure. Circulation (1963) 27:29–35. 14015085 10.1161/01.cir.27.1.29

[34] Arkles JS Opotowsky AR Ojeda J Rogers F Liu T Prassana V. Shape of the right ventricular Doppler envelope predicts hemodynamics and right heart function in pulmonary hypertension. Am J Respir Crit Care Med. (2011) 183:268–76. 10.1164/rccm.201004-0601OC 20709819

[35] Hussain N Ramjug S Hurdman J Elliot C Kiely D Non-invasive testing can help differentiate idiopathic pulmonary arterial hypertension and pulmonary hypertension associated with heart failure with preserved ejection fraction. Am J Respir Crit Care Med. (2014) 189:A1891 2014.189.1_MeetingAbstracts.A1891 10.1164/ajrccm-conference

[36] Afilalo J Grapsa J Nihoyannopoulos P Beaudoin J Gibbs JS Channick RN. Leaflet area as a determinant of tricuspid regurgitation severity in patients with pulmonary hypertension. Circ Cardiovasc Imaging (2015) 8: e002714. 10.1161/CIRCIMAGING.114.002714 25977303 PMC4435735

[37] Opotowsky AR Ojeda J Rogers F Prasanna V Clair M Moko L. A simple echocardiographic prediction rule for hemodynamics in pulmonary hypertension. Circ Cardiovasc Imaging (2012) 5:765–75. 10.1161/CIRCIMAGING.112.976654 22914595 PMC3505751

[38] D'Alto M Romeo E Argiento P Pavelescu A D'Andrea A Di Marco GM. A simple echocardiographic score for the diagnosis of pulmonary vascular disease in heart failure. J Cardiovasc Med. (2017) 18:237–43. 10.2459/JCM.0000000000000485 27841823

[39] Mooij CF de Wit CJ Graham DA Powell AJ Geva T. Reproducibility of MRI measurements of right ventricular size and function in patients with normal and dilated ventricles. J Magn Reson Imaging (2008) 28:67–73. 10.1002/jmri.21407 18581357 PMC2533688

[40] Marcus JT Vonk Noordegraaf A Roeleveld RJ Postmus PE Heethaar RM Van Rossum AC. Impaired left ventricular filling due to right ventricular pressure overload in primary pulmonary hypertension: noninvasive monitoring using MRI. Chest (2001) 119:1761–5 11399703 10.1378/chest.119.6.1761

[41] Sanz J Kariisa M Dellegrottaglie S Prat-González S Garcia MJ Fuster V. Evaluation of pulmonary artery stiffness in pulmonary hypertension with cardiac magnetic resonance. JACC Cardiovasc Imaging (2009) 2:286–95. 10.1016/j.jcmg.2008.08.007 19356573

[42] Swift AJ Rajaram S Condliffe R Capener D Hurdman J Elliot CA. Diagnostic accuracy of cardiovascular magnetic resonance imaging of right ventricular morphology and function in the assessment of suspected pulmonary hypertension results from the ASPIRE registry. J Cardiovasc Magn Reson. (2012) 14:40. 10.1186/1532-429X-14-40 22720870 PMC3419131

[43] Richter SE Roberts KE Preston IR Hill NS. A simple derived prediction score for the identification of an elevated pulmonary artery wedge pressure using precatheterization clinical data in patients referred to a Pulmonary Hypertension center. Chest (2016) 149:1261–8. 10.1378/chest.15-0819 26501213

[44] Braunwald E Frahm C Studies on Starling's law of the heart. IV. Observations on the hemodynamic functions of the left atrium in man. Circulation (1961) 24:633–42.

[45] Walston A 2nd Kendall ME. Comparison of pulmonary wedge and left atrial pressure in man. Am Heart J. (1973) 86:159–64. 4719936 10.1016/0002-8703(73)90239-1

[46] Robbins IM Hemnes AR Pugh ME Brittain EL Zhao DX Piana RN. High prevalence of occult pulmonary venous hypertension revealed by fluid challenge in pulmonary hypertension. Circ Heart Fail (2014) 7:116–22. 10.1161/CIRCHEARTFAILURE.113.000468 24297689 PMC3934572

[47] Fox BD Shimony A Langleben D Hirsch A Rudski L Schlesinger R. High prevalence of occult left heart disease in scleroderma-pulmonary hypertension. Eur Respir J. (2013) 42:1083–91. 10.1183/09031936.00091212 23258775

[48] D'Alto M Romeo E Argiento P Motoji Y Correra A Di Marco GM. Clinical relevance of fluid challenge in patients evaluated for pulmonary hypertension. Chest (2017) 151:119–26. 10.1016/j.chest.2016.08.1439 27575357

[49] Fujimoto N Borlaug BA Lewis GD Hastings JL Shafer KM Bhella PS. Hemodynamic responses to rapid saline loading: the impact of age, sex, and heart failure. Circulation (2013) 127:55–62. 10.1161/CIRCULATIONAHA.112.111302 23172838 PMC3549554

[50] Naeije R Vanderpool R Dhakal BP Saggar R Saggar R Vachiery J-L. Exercise-induced pulmonary hypertension. physiological basis and methodology concerns. Am J Respir Crit Care Med. (2013) 187:576–83. 10.1164/rccm.201211-2090CI 23348976 PMC3733438

[51] Maor E Grossman Y Balmor RG Segel M Fefer P Ben-Zekry S. Exercise haemodynamics may unmask the diagnosis of diastolic dysfunction among patients with pulmonary hypertension. Eur J Heart Fail (2015) 17:151–8. 10.1002/ejhf.198 25488133

[52] Califf RM Adams KF McKenna WJ Gheorghiade M Uretsky BF McNulty SE. A randomized controlled trial of epoprostenol therapy for severe congestive heart failure: The Flolan International Randomized Survival Trial (FIRST). Am Heart J. (1997) 134:44–54. 9266782 10.1016/s0002-8703(97)70105-4

[53] Kalra PR Moon JC Coats AJ. Do results of the ENABLE (Endothelin Antagonist Bosentan for Lowering Cardiac Events in Heart Failure) spell the end for non-selective endothelin antagonism in heart failure? Int J Cardiol. (2002) 85:195–97. 10.1016/S0167-5273(02)00182-1 12208583

[54] Bonderman D Ghio S Felix SB Ghofrani HA Michelakis E Mitrovic V. Riociguat for patients with pulmonary hypertension caused by systolic left ventricular dysfunction: a phase IIb double-blind, randomized, placebo-controlled, dose-ranging hemodynamic study. Circulation (2013) 128:502–11. 10.1161/CIRCULATIONAHA.113.001458 23775260

[55] Bermejo J Yotti R García-Orta R Sánchez-Fernández PL Castaño M Segovia-Cubero J. Sildenafil for improving outcomes in patients with corrected valvalar heart disease and persistent pulmonary hypertension: a multicenter, double-blind, randomized, controlled trial. Eur Heart J. (2018) 39:1255–64. 10.1093/eurheartj/ehx70 29281101 PMC5905634

